# Engineering an Industrial *Streptomyces albus* Strain to Enable High-Yield Heterologous Production of Spectinabilin

**DOI:** 10.3390/microorganisms14061201

**Published:** 2026-05-26

**Authors:** Xueyu Wang, Zhixing Gong, Jiaxiu Wei, Jianxin Dong, Wenjun Guan

**Affiliations:** 1 Department of Respiratory Medicine, The Fourth Affiliated Hospital, Zhejiang University School of Medicine, Yiwu 322000, China; 2 Polytechnic Institute of Zhejiang University, Hangzhou 310015, China

**Keywords:** *Streptomyces* host, spectinabilin, polyketide, biosynthetic gene cluster, heterologous biosynthesis

## Abstract

*Streptomyces* species are major producers of bioactive molecules via biosynthetic gene clusters (BGCs). However, many BGCs are silent or poorly expressed in their native hosts, making heterologous expression hosts a key strategy for discovering novel natural products and efficiently producing known compounds. In this study, *Streptomyces albus* ZD11, an industrial salinomycin producer capable of efficiently utilizing soybean oil to supply abundant polyketide precursors, was selected as a candidate host for the expression of polyketide BGCs. A genome-reduced derivative, designated ZD12, was constructed by deleting four endogenous polyketide BGCs from ZD11, aiming to reduce precursor competition and alleviate metabolic burden. To evaluate the polyketide biosynthesis capacity of ZD12, an engineered spectinabilin BGC was heterologously expressed in both ZD12 and a commonly used heterologous host *S. albus* J1074. The resulting ZD12-derived strain DHM produced 412 mg/L spectinabilin, while the J1074-derived strain J-DHM produced 114 mg/L, both of which were significantly higher than the native production level in *S. spectabilis*. Notably, the titer in DHM exceeded the highest previously reported heterologous titer by more than threefold. Furthermore, under identical integration conditions, DHM achieved a 2.6-fold higher spectinabilin titer than J-DHM, demonstrating the superior polyketide biosynthesis capacity of ZD12.

## 1. Introduction

*Actinomycetes*, particularly *Streptomyces* species, are well-known for their ability to produce a plethora of bioactive natural products, including polyketides, nonribosomal peptides, and β-lactams. Among these metabolites, polyketides are a class of secondary metabolites with structural diversity and wide clinical applications [1,2]. At present, a variety of reasonable metabolic engineering methods have been adopted to increase the production of valuable polyketides, such as overexpressing rate-limiting enzymes or transcriptional activators, increasing the supply of biosynthetic precursors, and heterologously expressing biosynthetic gene clusters (BGCs) in the appropriate hosts [3]. *Streptomyces* species offer the advantages of abundant precursor supply, accessible genetic engineering, and a growth rate conducive to efficient fermentation, making it widely regarded as the preferred host for producing polyketides [4]. Several *Streptomyces* strains, such as *Streptomyces coelicolor* A3(2), *Streptomyces lividans* TK24 and *Streptomyces albus* (syn. *Streptomyces albidoflavus*) [5] J1074, have been engineered as host for heterologous biosynthesis of many natural products [4,6].

*Streptomyces albus* ZD11 [7], a derivative of an industrial salinomycin-producing strain, has the potential to utilize soybean oil as the primary carbon source for the high-yield production of salinomycin (a polyether polyketide antibiotic). This strain could achieve salinomycin titer of up to 10 g/L in shake-flask cultures and 20 g/L under optimized bioreactor conditions [8]. Our previous studies have demonstrated that ZD11 leverages its inherently robust fatty acid β-oxidation pathway to generate abundant acyl-CoA precursors, thereby supporting efficient synthesis of polyketides [9,10]. Moreover, the relatively slow and stable utilization of oils extends the fermentation period, promoting the accumulation of secondary metabolites. Compared to sugar-based carbon sources (e.g., glucose, sucrose), oils possess a higher energy density. This allows ZD11 to derive more energy from less carbon input, supporting both growth and secondary metabolism. In our previous works, two polyketide BGCs, actinorhodin BGC (coloned from *S. coelicolor*) and daunorubicin BGC (coloned from *Streptomyces coeruleorubidus*) [9], were heterologously expressed in a ZD11 mutant Δsal, which lacks the native salinomycin BGC. When produced heterologously in Δsal, both daunorubicin and actinorhodin achieved higher titers than those obtained from their native strains (unpublished data). Consequently, ZD11 demonstrates significant potential as a superior heterologous host for constructing efficient cell factories for polyketide production.

Aureothin [11], alloaureothin [12], spectinabilin (or neoaureothin) [13], luteoreticulin (or griseulin) [14], and luteothin (or deoxyaureothin) [11] are five structurally related nitrophenyl pyrones isolated from *Streptomyces* species. The backbone of these compounds is assembled by type I polyketide synthase (PKS). Among them, spectinabilin is produced by both *Streptomyces spectabilis* and *Streptomyces orinoci*, differing from aureothin solely in the length of its polyketide chain. Spectinabilin and its derivatives were found to exhibit antiproliferative, antiplasmodial, antiviral, and nematocidal activities [15,16,17], indicating its extensive application prospect. The core biosynthetic pathway of spectinabilin had been largely elucidated (Figure S1) [11,18,19]. To increase the fermentation titer of spectinabilin, various strategies were employed, including heterologous expression of the BGC, targeted genetic engineering of the producer strain, and mutagenesis screening of native strain [20,21,22,23]. The spectinabilin BGC contains fourteen open reading frames (ORFs), which were designated *spnA-M* (Table S1) [21]. This BGC was heterologously expressed in *S. lividans* and *S. coelicolor*, resulting in a maximum spectinabilin titer of 105 mg/L among the recombinant strains [20].

In this study, in order to modify ZD11 into a host more suitable for expressing polyketide BGC, four endogenous polyketide BGCs were identified and knocked out in ZD11. The phenotype of resulting strain, ZD12, was then carefully characterized. Subsequently, the spectinabilin BGC from *S. spectabilis* was cloned, modified, and integrated into the chromosomes of both ZD12 and a commonly used heterologous host *S. albus* J1074 for comparison. Our finding indicates that ZD12 serves as a valuable host that can complement existing well-characterized heterologous hosts for natural product discovery and production.

## 2. Materials and Methods

### 2.1. Media and Cultivation Conditions

All strains and vectors used in this work are listed in Table S2. For cloning and vector amplification, *Escherichia coli* strains were cultured in Luria–Bertani (LB) medium supplemented with spectinomycin (50 mg/L), apramycin (50 mg/L), kanamycin (50 mg/L) or chloramphenicol (25 mg/L). For sporulation, *S. albus* ZD11 and *S. spectabilis* strains were grown on ISP4 (ISP Medium No. 4) agar plates (BD, Franklin Lakes, NJ, USA) for ten days; *S. albus* J1074 was grown on MS (Mannitol Soya Flour) agar plates (20 g/L soybean flour, 20 g/L mannitol, 20 g/L agar) for eight days. For fermentation, ZD11 and its derivatives were cultured in YMGD medium (10 g/L malt extract, 4 g/L yeast extract, 4 g/L glucose, 50 g/L dextrin, 1 g/L calcium carbonate, and 10% soybean oil) at 32 °C 180 rpm as previously reported [10]; *S. albus* J1074 and its derivatives were cultured in YEME-CaCO_3_ medium (40 g/L glucose, 3 g/L yeast extract, 3 g/L malt extract, 5 g/L tryptone, and 1 g/L calcium carbonate) at 30 °C 220 rpm; *S. spectabilis* was cultured in the liquid medium reported previously [16].

### 2.2. Isolation of DNA

All routine experiments such as DNA isolation, plasmid preparation, restriction digests, PCR, gel electrophoresis, ligation, and transformation were performed according to standardized methods for *E. coli* [24]. Isolation of genomic DNA from the *Streptomyces* strains was performed according to the standard procedure [25]. Genomic DNA was digested with *EcoRV*, precipitated with ethanol, washed twice with 70% ethanol (*v*/*v*), and air-dried. The DNA pellets were resuspended in 12 µL of autoclaved ddH_2_O.

### 2.3. RNA-Seq Analysis and RT-qPCR Assay

For total RNA extraction, *S. albus* ZD11 and its related mutants were grown in YMGD media. Three replicates of mycelia were collected at 24 h from the media and stored at −80 °C until RNA extraction. Total RNA isolation, RNA-seq and RT-qPCR were performed according to a previous report [9]. Technical triplicates of three biological repeats were performed for each condition.

### 2.4. Acquisition and Modification of the Spectinabilin Biosynthetic Gene Cluster

Two DNA receivers p48 and p45 were amplified using KOD FX (TOYOBO, Tokyo, Japan) polymerase following manufacturer’s protocol using plasmid pBE48 and pBE45 as template respectively [25]. Each DNA receptor has a homology of 39 bp with a fragment containing spectinabilin BGC. *E. coli* NEB10β cells harboring pBE14 circularization helper plasmid were prepared to electroporation for Cre-lox in vivo recombination [25]. The digested genomic DNA of *S. spectabilis* was assembled with p45 and p48, then electroporated into *E. coli* NEB10β, and transformants were plated on LB agar containing 50 mg/L apramycin. The primer pair pSPEC-con-F/R was used to select the correct assembly. The vector (named pSpec) carrying the correct assembly was purified using HiPure BAC DNA Mini Kits (Magen, Guangzhou, China).

Modification of the spectinabilin BGC was achieved through the PCR-targeting system. The promoter and upstream redundant sequence of *spnD* (56–9691 bp, a total of 9.5 kb) on the pSpec vector (75.6 kb) were replaced with kasOp*, resulting in the pSKD vector (66.9 kb). A redundant sequence downstream of *spnM* and the replication origin gene *ori2* (47,624–63,413 bp, a total of 15.8 kb) on the pSKD vector were replaced with a spectinomycin resistance gene and the replication origin *p15A ori*, resulting in the pSKD-SmR-p15A vector (53 kb). To construct the two-part system, a pSKD-derived vector pSKD-D-H (27.3 kb) was generated by replacing a 40.5 kb downstream fragment of *spnH* (18,635–59,115 bp) with a chloramphenicol resistance gene. A pSKD-derived vector pSKD-H-M was generated by replacing the sequences flanking the *spnH-spnM* region (35.6 kb total: regions 1–16,348 bp and 47,624–66,856 bp) with a hygromycin resistance gene, VWB integrase, and associated conjugative transfer elements. All vectors were verified by PCR and restriction enzyme digestion.

### 2.5. Construction and Evaluation of the BGC-Knockout Mutants

The Δars mutant was generated by deleting BGC-19 (*DUI70_3158–DUI70_3159*) in the ZD11 WT strain using a PCR-targeting knockout vector derived from BGC-19-harboring fosmid [8]. The Δcan, Δcon, or Δlas mutant was constructed in the ZD11 WT strain by markerless deletion of BGC-5 (*DUI70_0686–0695*), BGC-7 (*DUI70_0879–0893*), or BGC-9 (*DUI70_1051–1056*) via homologous recombination [26], respectively. The double-mutant ΔarsΔcon or ΔarsΔlas was generated from the Δars parental strain by deleting BGC-7 or BGC-9, respectively. Subsequently, the triple mutant ΔarsΔconΔcan and ΔarsΔconΔlas were constructed from the ΔarsΔcon background through markerless deletion of BGC-5 and BGC-9, respectively. Finally, the quadruple-mutant ΔarsΔconΔcanΔsal (named ZD12) and ΔarsΔconΔlasΔsal were derived from the ΔarsΔconΔcan and ΔarsΔconΔlas strains by markerless deletion of BGC-3 (*DUI70_0265–DUI70_0273*).

The fermentation process and analysis (salinomycin titer and biomass) of the abovementioned mutants were all carried out in accordance with the previously reported method [10].

### 2.6. Construction of Recombinant Strains Harboring the Spectinabilin BGC

The vectors carrying the spectinabilin BGC were integrated into *S. albus* via conjugation [27], followed by the selection of positive clones using the corresponding antibiotics. The vectors pSKD-D-H and pSKD-H-M were sequentially integrated into the φC31 and VWB sites of J1074 or ZD12, resulting in the recombinant strain J-DHM or DHM. The vector pSKD-SmR-p15A was integrated into the φC31 site of J1074 to generate the J-p15A mutant.

### 2.7. Cell Morphology Analysis

*S. albus* ZD12 were cultured on ISP4 agar plates at 30 °C for 6–8 days for collection of the mycelium or spore samples. Scanning electron microscopy (SEM) and cryo-SEM observation were performed at the Core Facilities of Zhejiang University School of Medicine. For SEM, samples were dried using an HCP-2 critical point dryer (Hitachi, Tokyo, Japan), sputter-coated with a conductive film, and examined with a scanning electron microscope Nova Nano 450 (Thermo FEI, Hillsboro, OR, USA). For cryo-SEM, samples were rapidly frozen in liquid nitrogen, transferred to a vacuum coater for sublimation at −90 °C for 8 min, followed by platinum sputter-coating at −120 °C. Imaging was performed using a focused ion beam scanning electron microscope Helios G3 (Thermo FEI, Hillsboro, OR, USA).

### 2.8. Secondary Metabolite Profiling

The profile of secondary metabolites produced by *S. albus* ZD11 and ZD12 strains were analyzed by high-performance liquid chromatography (HPLC). The fermentation broth was extracted overnight with methanol (1:9, *v*/*v*), followed by centrifugation and filtration through 0.22 μm organic membrane. The filtrate was analyzed on a ZORBAX Eclipse XDB-C18 HPLC column (Agilent, Santa Clara, CA, USA, 5 µm, 4.6 × 150 mm) using a water-acetonitrile gradient (solvent A: ultrapure water; solvent B: acetonitrile) at 1 mL/min. The elution program was as follows: 0–5 min, 5% B; 5–30 min, 5–100% B (linear); 30–35 min, 100% B; 35–36 min, 100–5% B; 36–40 min, 5% B. UV detection spanned 190–640 nm.

### 2.9. Analysis of Spectinabilin

Transfer 500 μL of fermentation broth to a 10 mL tube (pre-filled with 4.5 mL ethyl acetate). Sonicate for 10 min, stand to extract. After 12 h, centrifuge for 15 min at 9000 rpm. Transfer 1 mL supernatant to a new 2 mL tube. Freeze-dry, redissolve residue in methanol, filter (0.22 µm). Analyze 15 μL filtrate by reverse-phase HPLC (ZORBAX Eclipse XDB-C18 column, 4.6 × 150 mm, 5 μm) with gradient: 0–30 min, 25–90% B (linear); 30–35 min, 90–100% B (linear); 35–40 min, 100–25% B (linear), 40–42 min, 25% B at 0.8 mL/min (A: water/1% acetic acid; B: acetonitrile). Quantify spectinabilin (378 nm) via reference peak areas.

### 2.10. Ultra-Performance Liquid Chromatography–High-Resolution Mass Spectrometry (HPLC-HRMS)

UPLC-HRMS analysis was completed at the Analysis Center of Agrobiology and Environmental Sciences of Zhejiang University. Sample preparation was performed as described in the section of spectinabilin analysis.

LC: Waters UPLC (Waters Corp., Milford, MA, USA), ACQUITY UPLC HSS T3 column (Waters Corp. 1.8 μm, 2.1 × 150 mm) was used in all the chromatographic experiments. The mobile phase consisted of 0.1% formic acid in water (A) and 0.1% formic acid in acetonitrile (B), with a linear gradient program as follows: 0 min, 5% B; 25 min, 95% B; 28 min, 95% B; 29 min, 5% B. The flow rate was maintained at 0.3 mL/min, the column temperature at 50 °C, and the detection wavelengths were set at 320 nm. The injection volume was 3 μL.

MS: AB TripleTOF 6600^+^ System (AB SCIEX, Framingham, MA, USA). Mass-spectrometric detection was conducted in both positive and negative ionization modes over an *m*/*z* range of 100–2000 Da. The ion source parameters were set as follows: Gas 1 (Air) = 55 psi, Gas 2 (Air) = 55 psi, Curtain Gas (N2) = 35 psi, source temperature = 550 °C, and ion spray voltage = +5500 V (positive mode)/–4500 V (negative mode). For MS1 acquisition, the declustering potential (DP) was 80 V and collision energy (CE) was 10 V. MS/MS data were acquired in TOF MS–Product Ion–IDA (information-dependent acquisition) mode with a collision energy of ±40 ± 20 eV. Prior to analysis, the exact mass calibration was performed automatically before each analysis employing the Automated Calibration Delivery System.

## 3. Results

### 3.1. Construction of a Genome-Reduced Derivative of ZD11

As previously noted, *S. albus* ZD11 can supply ample acyl-CoA precursors via its robust fatty acid β-oxidation pathway, demonstrating strong potential as a universal host for polyketide biosynthesis. AntiSMASH [28] analysis of the ZD11 genome [10] revealed ten predicted polyketide BGCs (Table S3), including the known salinomycin BGC (BGC3). During secondary metabolite synthesis, these BGCs may not only consume substantial polyketide precursors but also complicate subsequent target compound identification and isolation. Therefore, selectively deleting non-essential endogenous polyketide BGCs in ZD11 will reduce precursor competition and create a genome-reduced host. This optimization should significantly enhance the efficiency of heterologous polyketide production in this host.

**Figure 1:**
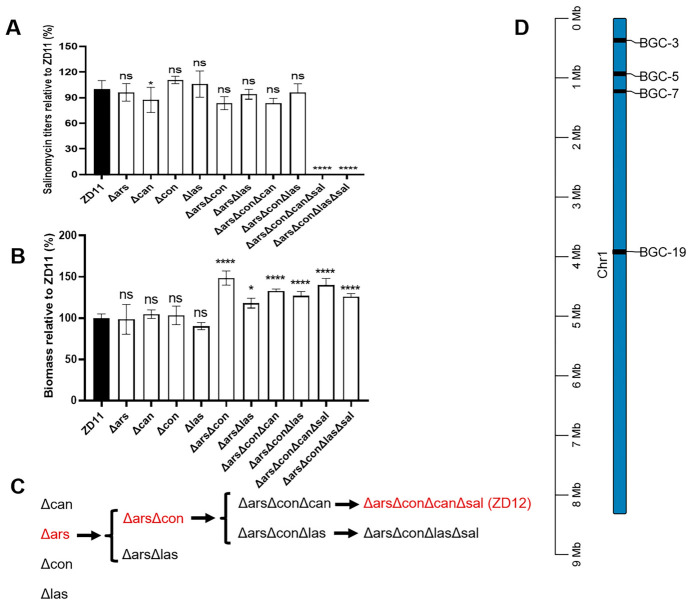
The fermentation results of the BGC-knockout mutants The salinomycin titers (**A**) and biomass (**B**) of different BGC-deletion mutants relative to ZD11 WT strain. (**C**) The construction process of ZD12. (**D**) The location of the knocked BGCs on the ZD12 genome. The symbols indicate the results of the significant differences analysis between the single BGC-knockout mutants and the ZD11 WT strain, ns: no significant difference, *p* ≥ 0.05; *: *p* < 0.05; ****: *p* < 0.0001. Fermentation medium: YMGD; fermentation time: 5 days.

Based on a comprehensive consideration of the total length of the BGCs and their transcriptional levels in the existing culture medium, we selected the top four ranked polyketide BGCs (BGC5, BGC7, BGC9, and BGC19; gray-highlighted in Table S3) to proceed to the knockout phase (Figure 1C). Due to antiSMASH’s limited predictive accuracy for BGC boundaries, this study targeted only the core genes within the selected BGCs. Finally, the Δars (ΔBGC19; 7.9 kb deletion), Δcan (ΔBGC5; 79.2 kb deletion), Δcon (ΔBGC7; 64.1 kb deletion) and Δlas (ΔBGC9; 47.8 kb deletion) mutants were generated. As shown in Figure 1, analysis of salinomycin titer and biomass revealed no significant differences versus the starting strain. Using Δars as the base strain, we sequentially knocked out BGC7 and BGC9 to create ΔarsΔcon and ΔarsΔlas. Compared with ZD11, these two mutants showed no difference in salinomycin titer but exhibited increased biomass. Given its superior biomass, ΔarsΔcon was selected for further engineering. Additional deletion of BGC5 and BGC9 in ΔarsΔcon generated the ΔarsΔconΔcan and the ΔarsΔconΔlas mutants, which also showed no difference in salinomycin titer but exhibited increased biomass levels compared to the ZD11 WT strain.

Both triple-deletion strains were subsequently used to knock out BGC3 (the salinomycin BGC; 78.2 kb deletion), generating ΔarsΔconΔcanΔsal mutant with 229.3 kb total deletions (2.8% of the ZD11 genome) and ΔarsΔconΔlasΔsal with 197.9 kb total deletions (2.4% of the ZD11 genome). As shown in Figure 1, shake-flask fermentation confirmed complete abolition of salinomycin production in both strains, while biomass increased to 140% and 126% of WT levels, respectively.

### 3.2. Characterization of the Genome-Reduced Mutant ZD12

**Figure 2:**
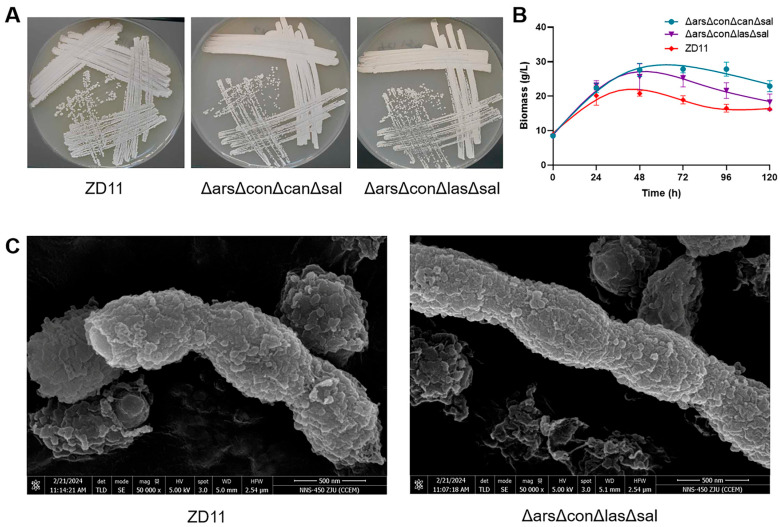
Growth phenotype of the ΔarsΔconΔcanΔsal and ΔarsΔconΔlasΔsal mutants. (**A**) Growth status on ISP4 plate. The strains were grown on an ISP4 plate for seven days. (**B**) The growth curves in the YMGD liquid medium. (**C**) Scanning electron microscope images of sporulating hyphae cultured on an ISP4 plate for six days. The scale bar is 500 nm.

As shown in Figure 2A,B, we examined the growth of ΔarsΔconΔcanΔsal and ΔarsΔconΔlasΔsal mutants in both solid and liquid media. The results indicate that deleting four polyketide BGCs did not significantly affect growth in the ISP4 plate. However, after 24 h of shake-flask fermentation, the biomass of both strains was significantly higher than that of the ZD11 WT strain. Due to its comparatively better growth, the ΔarsΔconΔcanΔsal mutant was consequently selected for subsequent research and designated ZD12. In addition, scanning electron microscopy revealed no significant differences in spore morphology and size between ZD12 and ZD11, suggesting that knockout of these BGCs did not affect the surface microstructure of ZD11 (Figure 2C).

**Figure 3:**
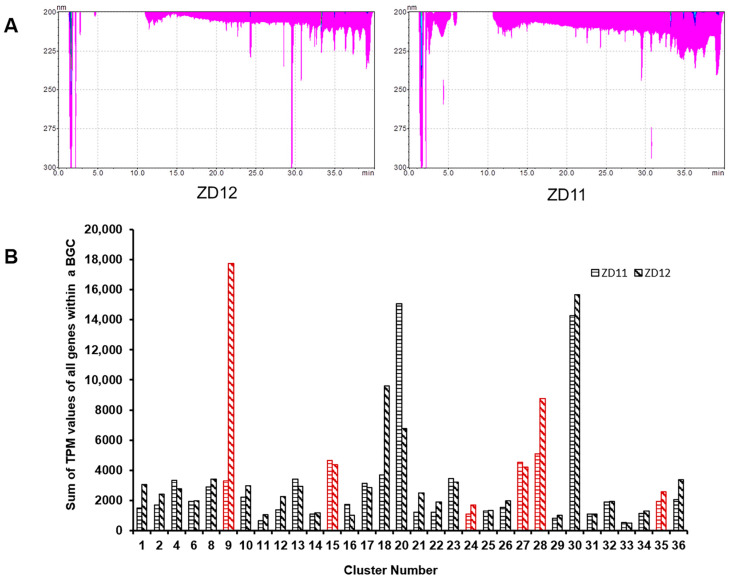
Comparison of the expression of endogenous BGCs in ZD12 and ZD11. (**A**) Iso-absorbance plots of the ZD12 and ZD11 strains. The methanol extract of fermentation broth is analyzed by HPLC with wavelength scanning from 200 nm to 300 nm. X-axis represents retention time (min) and Y-axis represents absorption wavelength (nm). (**B**) The total transcriptional level of each remaining BGC in ZD12 and ZD11. The numbers 1–36 on the X-axis correspond to the BGCs in ZD11 predicted by antiSMASH (Table S3). The red columns represent the predicted polyketide BGCs.

To compare the titer of endogenous secondary metabolites between ZD11 and ZD12, the strains were cultivated in a YMGD medium for five days. The metabolites were then extracted and analyzed using HPLC. Through wavelength scanning and iso-absorbance plot analysis, we observed that the main metabolite salinomycin (its absorption peak occurs at the intersection of 35–37.5 min and 200–210 nm) and several other metabolites had disappeared in ZD12 (Figure 3A). It indicates that ZD12 has a relatively cleaner and simpler metabolite profile compared to its parental strain ZD11, critically laying a solid foundation for the expression of heterologous polyketide BGCs.

To further investigate the physiological and metabolic consequences of quadruple BGC deletion in ZD11, the comparative transcriptome analysis was performed (NCBI accession number: PRJNA1273295). As shown in Figure S2A, relative to ZD11, the ZD12 mutant exhibited significant transcriptional alterations in 472 genes (7.2% of 6580 transcribed genes). Despite these changes, the core physiological functions remained largely unaffected. Specifically, 161 genes showed upregulation (log_2_FC > 1) while 311 were downregulated (log_2_FC < −1). KEGG enrichment analysis revealed predominant enrichment of upregulated genes in carbohydrate metabolism pathways, including citrate (TCA) cycle and carbon fixation, whereas downregulated genes were primarily associated with protein synthesis/catabolism and peptidoglycan biosynthesis/remodeling (Figure S2B,C). Notably, these transcriptomic changes correlate with the improved growth of ZD12, as well as the reduction in protein synthesis caused by knocking out four BGCs, especially the very high-expression salinomycin BGC (Figure 2B). This suggests that the quadruple BGC deletion reduces the metabolic burden in ZD12, thus enhancing biomass accumulation.

To investigate the impact of knocking out four polyketide BGCs on the transcription of the remaining BGCs in ZD12, we calculated the total transcriptional level of each BGC using transcriptome data (sum of TPM values for all genes within a BGC). Compared to ZD11, ZD12 showed significant upregulation of BGC1, 9, 18, and 21 (log_2_FC > 1) and downregulation of BGC20 (log_2_FC < −1), while the other 31 remaining BGCs exhibited no significant changes (Figure 3B). As previously mentioned, ZD11 contains ten predicted polyketide BGCs (Table S3). After knocking out four polyketide BGCs, only BGC9 displayed significantly increased transcription in ZD12, with no broad regulatory changes observed in the other five remaining polyketide BGCs. This finding demonstrates that the biosynthetic precursors conserved through the deletion of endogenous BGCs do not universally activate the expression of the remaining endogenous BGCs. In the absence of heterologous overexpression of a specific polyketide BGC, these conserved acyl-CoA precursors exhibit a preferential flux toward primary metabolic pathways. This observation implies that the activation of endogenous secondary metabolite BGCs is not contingent solely upon the accumulation of their biosynthetic precursors but rather involves the integrated actions of additional biochemical and physiological factors, which are still unclear.

### 3.3. Acquisition and Modification of the Spectinabilin BGC

**Figure 4:**
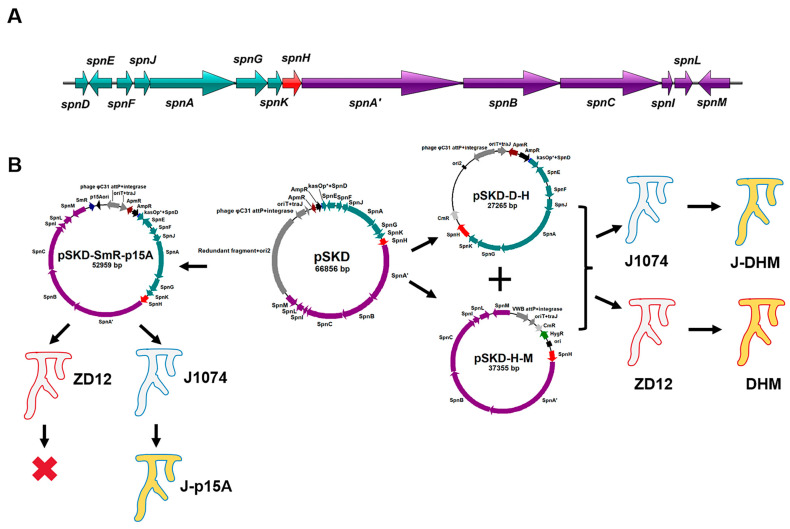
Construction of the vectors carrying spectinabilin BGC and their related mutant strains. (**A**) The composition of spectinabilin BGC. (**B**) Schematic of the construction of vectors carrying the spectinabilin BGC and the mutant expressing the spectinabilin BGC. Symbol “X” indicates that the target strain was not obtained.

Due to the diverse activities of spectinabilin and its derivatives, this study chose to heterologously express the spectinabilin BGC in ZD12 to evaluate its suitability as a polyketide host. The genome of *S. spectabilis* strain (purchased from CGMCC, Table S2) was sequenced to identify the spectinabilin BGC (Figure 4A). As shown in Figure 4B, using a published method [29], the spectinabilin BGC was cloned from *S. spectabilis* and modified to generate the vector pSKD, which contained the BGC flanked by 12.1 kb downstream region, the conjugation element, and the φC31 integrase gene [19]. In pSKD, the native promoter of pathway-specific regulator gene *spnD* within the spectinabilin BGC was replaced by the strong constitutive promoter kasOp* [30]. Finally, confirming the BGC boundaries (*spnD* to *spnM*) [21], the downstream 11.5 kb region and the low-copy *ori2* in pSKD were replaced by the fragment SmR-p15A (carrying the high-copy p15A *ori*), yielding the vector pSKD-SmR-p15A.

The pSKD-SmR-p15A vector could be readily integrated into the chromosome of J1074 (generating the J-p15A mutant strain). However, repeated attempts to introduce this vector into ZD12 were unsuccessful. This failure was likely due to the relatively low conjugative transfer efficiency for large vectors; an inherent property inherited from the industrial strain ZD11. To resolve this problem, the pSKD vector was then split into two smaller vectors: pSKD-D-H and pSKD-H-M. As shown in Figure 4B, pSKD-D-H contains the *spnD*-*spnH* genes and the original conjugation elements of pSKD, while pSKD-H-M contains the *spnH*-*spnM* genes, VWB integrase gene [19], and the associated conjugation elements. Finally, these two vectors were successfully integrated into the φC31 and VWB *attB* sites of ZD12, respectively, generating the DHM mutant. For comparison, pSKD-D-H and pSKD-H-M were also integrated into the same *attB* sites of *S. albus* J1074, creating the J-DHM mutant.

### 3.4. Heterologous Expression of the Spectinabilin BGC in ZD12 and J1074

**Figure 5:**
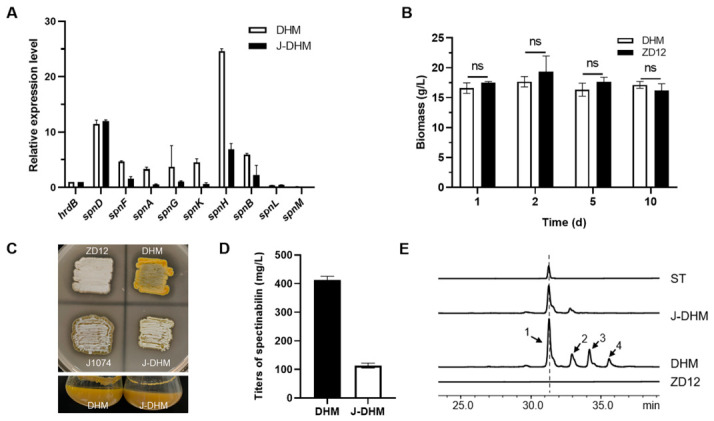
Heterologous expression of the spectinabilin BGC. (**A**) Expression levels of partial genes within the spectinabilin BGC in DHM and J-DHM (24 h), with *hrdB* used as reference gene. (**B**) The biomass of DHM and ZD12 (YMGD liquid medium). The symbol indicates the results of a significant difference analysis between DHM and ZD12, ns: no significant difference, *p* ≥ 0.05. (**C**) The growth status of DHM or J-DHM on ISP4 or MS plate. (**D**) The spectinabilin titers of DHM and J-DHM after 10-day shake-flask fermentation (YMGD liquid medium). (**E**) HPLC spectrum of the fermentation extract after 10 days of fermentation, measured at a wavelength of 378 nm. ST represents spectinabilin standard.

After obtaining the J-DHM and DHM mutants, we then analyzed the transcriptional levels of eight key genes in the spectinabilin BGC using RT-qPCR. As shown in Figure 5A, the transcriptional levels of *spnA*, *spnB*, *spnF*, *spnG*, *spnK*, and *spnH* were higher in DHM than those in J-DHM, while *spnM* was not expressed, suggesting that *spnM* may not be regulated by the pathway-specific regulator SpnD. The transcriptional levels of *spnD* and *spnL* were comparable in both strains. Notably, *spnH* exhibited significantly higher transcriptional level than other genes due to having two copies.

As shown in Figure 5C, after 6-day growth on ISP4 plate, the lawns of DHM and J-DHM appeared yellow. In addition, DHM exhibited sparser aerial hyphae than ZD12, while J-DHM’s resembled J1074’s. After ten days of flask-shaking in a YMGD medium, DHM and ZD12 showed comparable biomass (Figure 5B), indicating that spectinabilin biosynthesis does not significantly affect growth. In the YMGD medium, DHM produced 412 mg/L spectinabilin, the highest reported titer for heterologous spectinabilin biosynthesis. In contrast, J-DHM yielded only 114 mg/L in YEME-CaCO_3_ medium (the optimized medium for J1074 to produce spectinabilin), which is significantly lower than that of DHM (Figure 5D). These results highlight the superior polyketide production capacity of ZD12.

As shown in Figure 5E, HPLC analysis indicated that peak 1 is spectinabilin. Relative to J-DHM, the fermentation extract of DHM showed an increased concentration of peak 2, along with the appearance of new peaks 3 and 4. These peaks were all absent in ZD12, suggesting peak 3 and 4 might be the spectinabilin derivatives from heterologous BGC expression in ZD12. To analyze the structural information of these molecules, UPLC-HRMS was performed on extracts of the 10-day fermentation culture of DHM. Combined with the aforementioned HPLC analysis, UV spectra, and base peak chromatograms, this enabled the determination of the corresponding absorption peaks 1–4. As shown in Figure S3, due to natural isotope effects (^13^C), all mass spectra for peaks 1–4 exhibited double-peak characteristics. Peak 1 showed ion peaks at *m*/*z* 478.2/479.2 [M+H]^+^ with a predicted molecular formula of C_28_H_31_NO_6_, corresponding to spectinabilin. Peak 2 had ions at *m*/*z* 422.2/423.2 [M+H]^+^ (predicted C_25_H_27_NO_5_), lacking two -CH_2_- units compared to the substrate of SpnI, this is believed to result from the loss of one malonyl-CoA extension unit during polyketide chain elongation. Peak 3 exhibited ions at *m*/*z* 464.2/465.2 [M+H]^+^ (predicted C_28_H_33_NO_5_), consistent with the substrate of SpnH and inferred to be an intermediate of spectinabilin synthesis. Its accumulation in DHM may relate to insufficient SpnH catalytic activity in ZD12. Peak 4 showed ions at *m*/*z* 478.3/479.3 [M+H]^+^ (predicted C_29_H_35_NO_5_), containing one additional -CH_2_- unit versus Peak 3’s compound (C_28_H_33_NO_5_), and is inferred to be an intermediate resulting from methylation of SpnH’s substrate (i.e., Peak 3’s compound) (Figure S3) [21]. In summary, the generation mechanism of these non-target products requires further elucidation.

## 4. Discussion and Conclusions

Leveraging ZD11’s efficient soybean oil utilization and robust β-oxidation pathway, which converts fatty acids into acyl-CoA and ATP to provide ample precursors and energy for polyketide synthesis, this strain demonstrates strong potential as a universal polyketide host. In this study, we engineered ZD11 into the genome-reduced ZD12 by deleting four endogenous polyketide BGCs in order to minimize precursor diversion and metabolic burden. Growth phenotyping and comparative transcriptomics confirmed ZD12’s streamlined metabolic profile and enhanced growth rate. To evaluate ZD12’s polyketide compatibility, we heterologously expressed an engineered spectinabilin BGC in both ZD12 and J1074. Fermentation titers in DHM (412 mg/L) and J-DHM (114 mg/L) substantially exceeded native production in *S. spectabilis* (52 mg/L, Figure S4), and DHM surpassed the highest reported heterologous titer over 3-fold. Crucially, under identical integration conditions, DHM outperformed J-DHM 2.6-fold on spectibabilin titer, demonstrating that ZD12 has superior polyketide biosynthesis capacity.

Although ZD11 exhibits significant advantages in efficiently utilizing oils to generate abundant acyl-CoA precursors, its origin as an industrial strain results in considerably lower genetic manipulation efficiency compared to the commonly used *Streptomyces* hosts (e.g., *S. coelicolor* A3(2), *S. lividans* TK24, *S. albus* J1074). Industrial *Streptomyces* strains are typically selected for high-yield secondary metabolite production under fermentation conditions, often at the expense of genetic amenability, which is a trade-off that is to some extent universal [31]. As a widely used host, J1074 has undergone extensive laboratory domestication, resulting in the loss of restriction–modification systems (e.g., SalI deficiency), along with advantageous features such as a small genome and fast growth, all of which facilitate genetic manipulation [32]. In contrast, ZD11 likely retains a robust restriction–modification system, thereby posing a fundamental obstacle to standard genetic engineering approaches.

To date, none of the commonly used replicative vectors in *Streptomyces*, including pIJ101- and pKC1139-based derivatives, can be stably maintained in ZD11 [26]. As a direct consequence, all genome-editing tools that rely on autonomously replicating vectors, most notably the CRISPR-Cas9 system which typically requires a replicating vector for constitutive Cas9 expression and guide RNA delivery, fail to function in ZD11. Even attempts to use integrative vectors (e.g., φC31- or φBT1-based integration) may encounter low efficiency due to poor conjugal transfer in this strain. This technical impasse severely hinders efforts to repurpose ZD11 from a native producer into a clean, high-performance chassis for programmable polyketide biosynthesis; especially because systematic deletion of competing or redundant BGCs is a prerequisite for achieving predictable and high-titer production of target polyketides.

To circumvent the dependence on replicative vectors, we previously developed a markerless knockout system applicable to ZD11 [26]. This system employs an episomal vector-independent strategy, enabling successful deletion of four large DNA fragments (10–200 kb) from three industrial *Streptomyces* strains while reducing operation time by approximately 25% relative to conventional methods. However, the operational efficiency of this system still requires further improvement. Given that ZD11 likely harbors multiple endogenous polyketide BGCs that compete for precursor supply, eliminating these clusters one by one using the current method would be prohibitively time-consuming. Therefore, urgent improvements to the existing system are necessary, such as incorporating positive–negative selection markers, optimizing homologous arm design, or transiently expressing recombinases to enhance homologous recombination efficiency.

Another major limitation is the low conjugative transfer efficiency of this strain. Low conjugation efficiency is a common issue in *Streptomyces*. For example, it was reported that *Streptomyces iranensis* DSM 41954 exhibited such poor conjugative transfer efficiency that no transconjugants could be obtained using a suicide vector [33]. To address this problem, several strategies have been explored to improve conjugation efficiency. For instance, introducing the traJ gene into mobilizable plasmids increased the number of transconjugants in *S. coelicolor* by 10- to 100-fold [34]. In another study, heterologous expression of teichoic acid biosynthesis genes from an industrial strain led to up to a 1300-fold increase in conjugation efficiency in *Streptomyces hygroscopicus* [35]. In addition, the use of a replicative targetable vector system, such as pDS0007, allows transconjugants to be obtained even in strains with extremely poor conjugation efficiency, while also providing continuous selection for subsequent double-crossover events [33].

Collectively, the interconnected challenges facing ZD11, including the low frequency of homologous recombination, the lack of stable replicative vectors, the incompatibility with the CRISPR system, and the poor conjugation transfer efficiency, highlight an urgent need to develop efficient, strain-specific genetic manipulation systems for this strain. As noted in recent reviews [31], although synthetic biology strategies have greatly improved the genetic manipulability of *Streptomyces* in recent years, limitations due to strain-specific and design-specific factors still exist. Therefore, future studies should investigate these issues in depth. Ultimately, building a comprehensive genetic toolbox for ZD11 will not only unlock its potential as a high-performance chassis for polyketide production but also provide a reference example for converting other industrially important but genetically difficult *Streptomyces* strains into easy-to-manipulate synthetic biology platforms.

## Data Availability

The original contributions presented in this study are included in the article/Supplementary Material. Further inquiries can be directed to the corresponding author.

## Supplementary Materials

The following supporting information can be downloaded at: https://www.mdpi.com/article/10.3390/microorganisms14061201/s1, Figure S1: Proposed model for spectinabilin biosynthesis; Figure S2: (A) Volcano map of DEG analysis in RNA-seq. KEGG pathway enrichment analysis of the upregulated genes (B) and downregulated genes (C); Figure S3: Mass spectra of compounds corresponding to HPLC peaks 1–4 (Figure 5E) in DHM fermentation extracts; Figure S4: The spectinabilin titers of *S. spectabilis* in shake-flask culture at different time-point. Table S1: Annotation of the spectinabilin BGC; Table S2: Strains and vectors used in this study; Table S3: Biosynthetic gene clusters predicted in ZD11 by antiSMASH.
