# Supplementary material for: Engineering an Industrial Streptomyces albus Strain to Enable High-Yield Heterologous Production of Spectinabilin
Source: Microorganisms. 2026 May 26;14(6):1201. doi: 10.3390/microorganisms14061201 (PMC13304415; doi:10.3390/microorganisms14061201)
Supplement: Supplementary file 1 [file microorganisms-14-01201-s001.zip › microorganisms-4296272-supplementary.pdf]

Supplementary information

Engineering an Industrial *Streptomyces albus* Strain to Enable High-Yield Heterologous Production of Spectinabilin

Xueyu Wang [1], Zhixing Gong [2], Jiaxiu Wei [1], Jianxin Dong [1] and Wenjun Guan [1,*]

[1] Department of Respiratory Medicine, The Fourth Affiliated Hospital, Zhejiang University School of Medicine, Yiwu 322000, China

[2] Polytechnic Institute of Zhejiang University, Hangzhou 310015, China

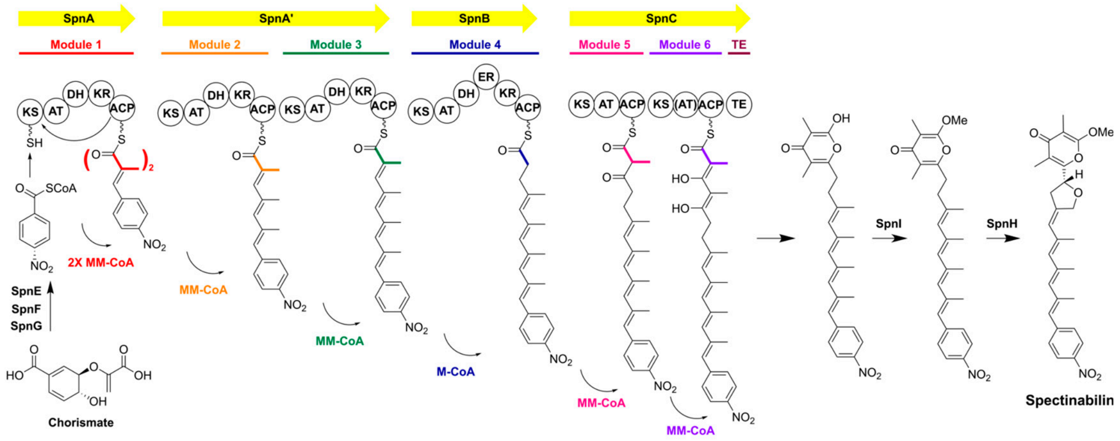

Figure S1. Proposed model for spectinabilin biosynthesis

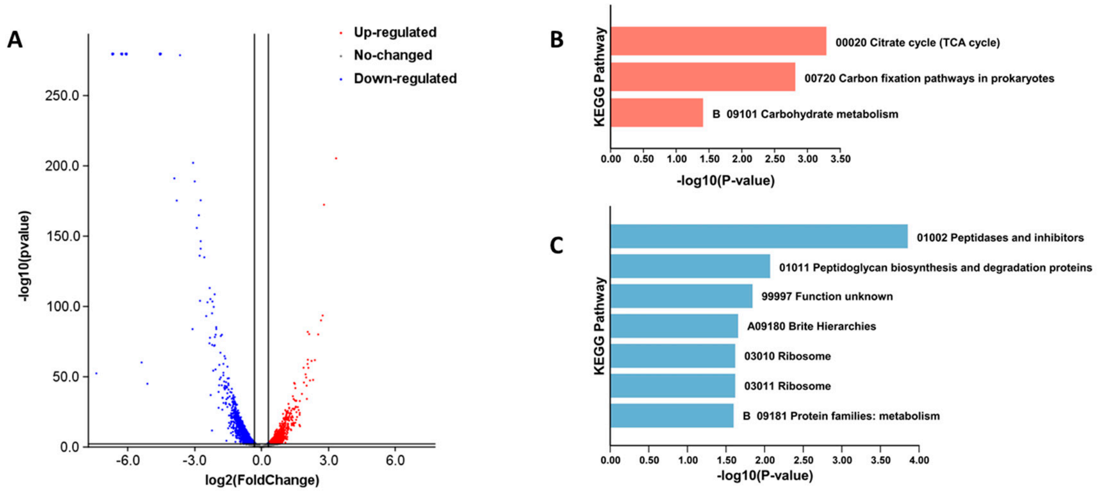

Figure S2. (A) Volcano map of DEG analysis in RNA-seq. Volcano plot displays the regulated transcripts in the ZD11 compared with those in the ZD12. KEGG pathway enrichment analysis of the upregulated genes (B) and downregulated genes (C).

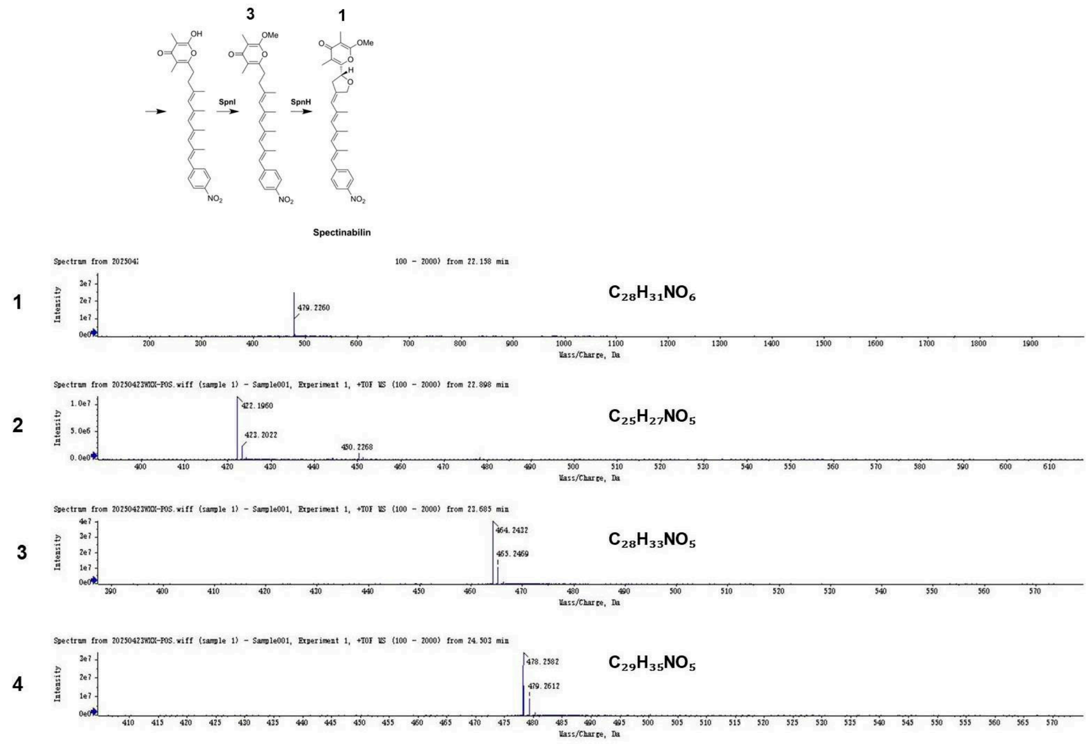

Figure S3. Mass spectra of compounds corresponding to HPLC peaks 1-4 (Fig. 5E) in DHM fermentation extracts.

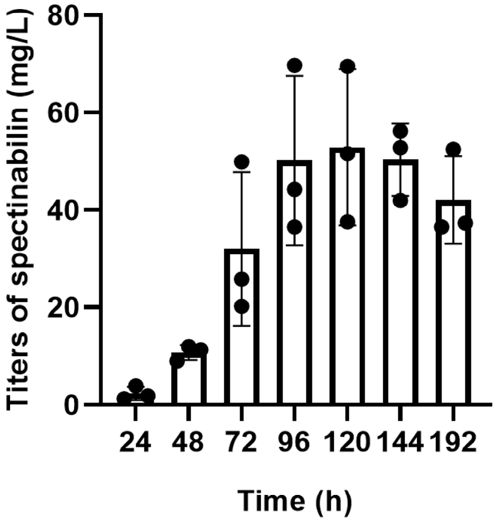

Figure S4. The spectinabilin titers of *S. spectabilis* in shake-flask culture at different time-point

Table S1. Annotation of the spectinabilin BGC

Gene	Gene locus			Direction of transcription	Distance from the previous gene (bp)	Proposed function
	From	To	Length (bp)			
spnD	1	840	840	+	/	Transcriptional activator
spnE	930	2447	1518	-	89	Fatty-acyl-CoA synthase
spnF	2821	3855	1035	+	373	Diiron oxygenase
spnJ	4023	5060	1038	+	167	3-deoxy-7-phosphoheptulonate synthase
spnA	5057	10831	5775	+	-4	Polyketide synthase
spnG	10913	13006	2094	+	81	Para-aminobenzoate synthetase
spnK	13054	13872	819	+	47	NA
spnH	14053	15285	1233	+	180	Cytochrome P450
spnA'	15352	26256	10905	+	66	Polyketide synthase
spnB	26243	32824	6582	+	-14	Polyketide synthase
spnC	32862	39701	6840	+	37	Polyketide synthase
spnI	39730	40431	702	+	28	Methyltransferase
spnL	40597	41784	1188	+	165	Acetyl-CoA acyltransferase
spnM	42197	44308	2112	-	412	MFS transporter

Table S2. Strains and vectors used in this study

Stains or vectors	Description	Reference
**Strains**		
E. coli TG1	host strain for DNA clone	Stratagene
E. coli DH5α	host strain for DNA clone	Stratagene
E. coli DH10B	host strain for DNA clone	Stratagene
E. coli ET12567 (pUZ8002)	dam- dcm- strain containing helper plasmid pUZ8002, Kan^R, Chl^R	Laboratory preservation
E. coli ET12567 (pUB307)	dam- dcm- strain containing helper plasmid pUB307, Kan^R, Chl^R	Laboratory preservation
S. albus J1074	naturally minimised *Streptomyces* strain	Laboratory preservation
S. spectabilis	harboring spectinabilin BGC	CGMCC：4.6311
S. albus ZD11	A derivative of salinomycin industrial strain	CGMCC 4.7658
Δars	ZD11 with disruption of BGC19 (*DUI70_3158* - *DUI70_3159*)	This study
Δcan	ZD11 with disruption of BGC5 (*DUI70_0686* - *DUI70_0695*)	This study
Δcon	ZD11 with disruption of BGC7 (*DUI70_0879* - *DUI70_0893*)	This study
Δlas	ZD11 with disruption of BGC9 (*DUI70_1051* - *DUI70_1056*)	This study
ΔarsΔcon	Δars with disruption of BGC7	This study
ΔarsΔlas	Δars with disruption of BGC9	This study
ΔarsΔconΔcan	ΔarsΔcon with disruption of BGC5	This study
ΔarsΔconΔlas	ΔarsΔcon with disruption of BGC9	This study
ΔarsΔconΔcanΔsal	ΔarsΔconΔcan with disruption of BGC3	This study
ΔarsΔconΔlasΔsal	ΔarsΔconΔlas with disruption of BGC3	This study
J-DHM	J1074 harboring vector pSKD-D-H and pSKD-H-M, $Apra^R$, $HygB^R$	This study
DHM	ZD12 harboring vector pSKD-D-H and pSKD-H-M, $Apra^R$, $HygB^R$	This study
**Vectors**		
9E4ars-fosmid-scar	A BGC19 knockout vector derived from a fosmid containing BGC19, $Apra^R$	This study

**Stains or vectors**	**Description**	**Reference**
pSUC-canHR	The BGC5 knockout vector derived from pSUC01 by inserting 3001 bp left arm and 3010 bp right arm of BGC5 into the *EcoRI/KpnI* sites, $Apra^R$	This study
pSUC-conHR	The BGC7 knockout vector derived from pSUC01 by inserting 3036 bp left arm and 3083 bp right arm of BGC7 into the *EcoRI/KpnI* sites, $Apra^R$	This study
pSUC-lasHR	The BGC9 knockout vector derived from pSUC01 by inserting 2971 bp left arm and 2976 bp right arm of BGC9 into the *EcoRI/KpnI* sites, $Apra^R$	This study
pSUC-salHR	The BGC3 knockout vector derived from pSUC01 by inserting 3047 bp left arm and 3013 bp right arm of BGC3 into the *EcoRI/KpnI* sites, $Apra^R$	This study
pSpec	A vector carring the spectinabilin BGC, the elements for conjugative transfer and φC31 integrase gene, $Apra^R$	This study
pSKD	The promoter of *spnD* in pSpec was replaced by kasOp*, $Apra^R$	This study
pSKD-SmR-p15A	Replace the redundant genes and replicator in pSKD with the spectinomycin resistance gene and the p15A *ori*, SpecR, ApraR	This study
pSKD-D-H	Carrying *spnD* – *spnH* from the spectinabilin BGC, the elements for conjugative transfer, and φC31 integrase gene, $Apra^R$	This study
pSKD-H-M-hyg	Carrying *spnH* – *spnM* from the spectinabilin BGC, the elements for conjugative transfer, and VWB integrase gene, Chl^R, $HygB^R$	This study

Table S3. Biosynthetic gene clusters predicted in ZD11 by antiSMASH

BGC	Type	Gene ID
**1**	NRPS	*DUI70_0110 - DUI70_0148*
**2**	NRPS	*DUI70_0173 - DUI70_0214*
**3**	T1PKS	*DUI70_0241 - DUI70_0289*
**4**	2dos(2-deoxy-streptamine aminoglycoside)	*DUI70_0576 - DUI70_0611*
**5**	T1PKS, NRPS-like, PKS-like	*DUI70_0662 - DUI70_0718*
**6**	terpene, RiPP-like	*DUI70_0757 - DUI70_0785*
**7**	T1PKS, NRPS-like	*DUI70_0861 - DUI70_0912*
**8**	LAP, thiopeptide	*DUI70_0919 - DUI70_0942*
**9**	T1PKS, terpene	*DUI70_1033 - DUI70_1077*
**10**	terpene, lanthipeptide-class-I, NRPS	*DUI70_1086 - DUI70_1151*
**11**	lanthipeptide-class-i, phosphonate	*DUI70_1163 - DUI70_1194*
**12**	NI-siderophore	*DUI70_1437 - DUI70_1459*
**13**	other	*DUI70_1909 - DUI70_1947*
**14**	terpene	*DUI70_2032 - DUI70_2048*
**15**	T1PKS	*DUI70_2157 - DUI70_2192*
**16**	lassopeptide	*DUI70_2222 - DUI70_2243*
**17**	butyrolactone	*DUI70_2705 - DUI70_2715*
**18**	melanin	*DUI70_3021 - DUI70_3030*
**19**	T1PKS, PKS-like	*DUI70_3134 - DUI70_3177*
**20**	arylpolyene, NRPS-like, NRP-metallophore, NRPS	*DUI70_3247 - DUI70_3331*
**21**	NRPS-like, betalactone	*DUI70_3814 - DUI70_3843*
**22**	lanthipeptide-class-iii	*DUI70_3878 - DUI70_3901*
**23**	NAPAA	*DUI70_4256 - DUI70_4276*
**24**	PKS-like, butyrolactone	*DUI70_4572 - DUI70_4607*
**25**	lanthipeptide-class-iii	*DUI70_4753 - DUI70_4771*

BGC	Type	Gene ID
**26**	NRPS-like,arylpolyene, other	*DUI70_4808 - DUI70_4867*
**27**	hglE-KS, NRPS-like, T2PKS, betalactone	*DUI70_4972 - DUI70_5062*
**28**	T1PKS	*DUI70_5419 - DUI70_5454*
**29**	RiPP-like	*DUI70_5540 - DUI70_5546*
**30**	arylpolyene,melanin	*DUI70_5551 - DUI70_5593*
**31**	ectoine	*DUI70_5897 - DUI70_5905*
**32**	terpene	*DUI70_6681 - DUI70_6701*
**33**	nucleoside	*DUI70_6732 - DUI70_6753*
**34**	betalactone	*DUI70_6800 - DUI70_6838*
**35**	T1PKS, NRPS	*DUI70_6851 - DUI70_6902*
**36**	NI-siderophore	*DUI70_7106 - DUI70_7132*
